# Data on the recovery of glycinergic neurons after spinal cord injury in lampreys

**DOI:** 10.1016/j.dib.2019.105092

**Published:** 2020-01-03

**Authors:** Silvia María Valle-Maroto, Antón Barreiro-Iglesias, Blanca Fernández-López, María Celina Rodicio

**Affiliations:** Department of Functional Biology, CIBUS, Faculty of Biology, Universidade de Santiago de Compostela, 15782, Santiago de Compostela, Spain

**Keywords:** Glycine, Neuronal regeneration, Spinal cord injury, Neurotransmitter, Central nervous system repair

## Abstract

We used immunohistochemical methods to quantify changes in the number of glycine-immunoreactive neurons of the dorsomedial, lateral and cerebrospinal fluid contacting cell populations of the spinal cord of larval sea lampreys after a complete spinal cord injury. The data presented here are quantifications of the number of glycine-immunoreactive neurons located in the rostral and caudal stumps of the spinal cord and the corresponding statistical analyses. These data show that, glycine immunoreactivity is lost in glycinergic neurons immediately after injury and that the number of glycine-immunoreactive neurons is recovered in the following two weeks. These data are useful for researchers investigating determinants that underlie the spontaneous recovery of locomotion following spinal injuries in regenerating animal models, and for analysing the role of glycinergic neurons in spinal cord repair after an injury.

**Table:** Specifications Table

Subject	Neuroscience
Specific subject area	Regenerative Biology, spinal cord injury and neuroplasticity
Type of data	Tables Graph Figures
How data were acquired	Spectral confocal microscope (TCS-SP2; Leica, Wetzlar, Germany) Softwares: LCS Lite (Leica), Fiji (Image J, NIH, Bethesda, Maryland, USA), Prism 6 (GraphPad software, La Jolla, CA), Photoshop CC 2018 (Adobe Inc., San José, CA), Corel Draw 2018 (Corel, Ottawa, Canada).
Data format	Raw Analysed
Parameters for data collection	Spinal cord injury: complete transection of the larval sea lamprey spinal cord at the level of the 5th gill. Experimental groups: control non-injured, n = 9; injured animals, n = 24 Recovery time points after injury: 1 hour (n = 9), 2, 4 and 10 weeks (n = 5 in each group). Images taken at 20× magnification with 1.5× digital zoom, within 150–300 μm rostral and caudal to the site of injury, or an equivalent region in control animals.
Description of data collection	At the time of processing, we took the region of the body comprised between the 4th and the 6th gills and fixed it in 5% glutaraldehyde and 1% sodium metabisulfite in 0.05 M TBS. The tissue was then sectioned in a cryostat at 14 μm, and we performed glycine immunofluorescence on the sections.
Data source location	Institution: Department of Functional Biology, Faculty of Biology, CIBUS, Universidade de Santiago de Compostela City: Santiago de Compostela Country: Spain
Data accessibility	With the article

**Table:** 

**Value of the Data** • These data could be compared with data on the recovery of other neurotransmitter systems, both in regenerating and non-regenerating vertebrates, to understand how different systems adapt to the post-injury situation. • This data set is of interest for researchers studying the factors that influence the spontaneous restoration of locomotion after spinal cord injury in regenerating animal models. • This data set is of interest for the investigation of the role of glycinergic neurons in recuperation from spinal cord injury, providing valuable information to find new ways to facilitate recovery from spinal cord injury in non-regenerating animal models.

## 1 Data

**Fig. 1:**
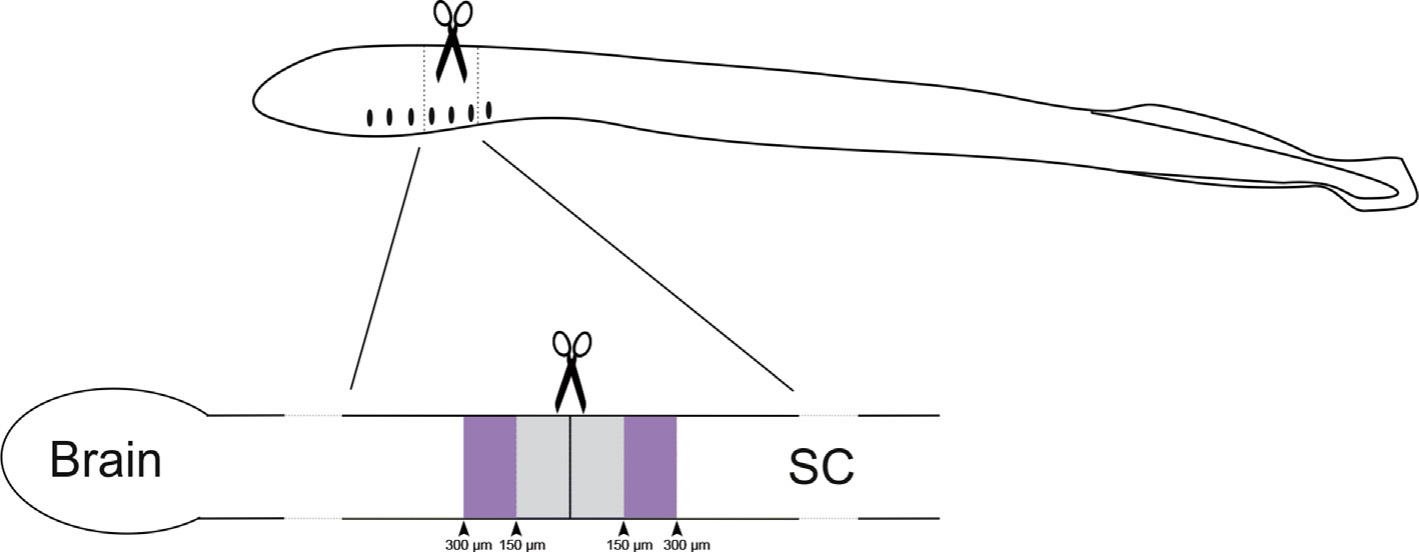
Schematic representations of a lateral view of a larva indicating the site of injury (scissors) and a lateral view of the spinal cord (SC) indicating the analysed areas (purple). Drawing modified from Fernández-López et al., 2016 [1], under a CC-BY 4.0 license.

**Fig. 2:**
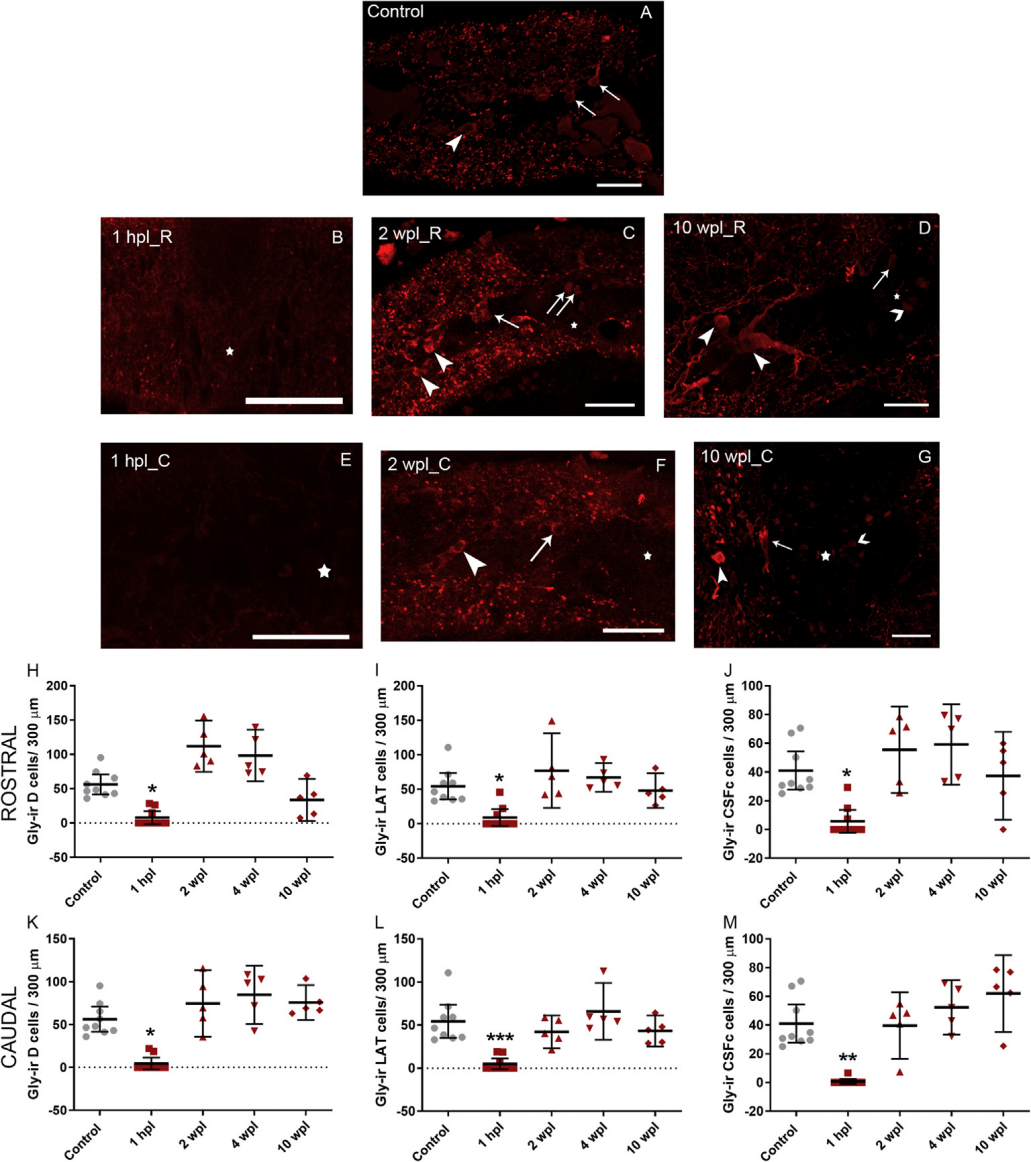
A–G: Confocal photomicrographs of transverse sections of the spinal cord showing glycine-ir neurons in control animals (A) and in the rostral (B–D) and caudal (E–G) stumps of the cord of 1 hpl (B, E), 2 wpl (C, F) and 10 wpl (D, G) larvae. H–M: Graphs showing changes in the number of glycine-ir dorsal interneurons (H and K), lateral interneurons (I and L) and CSFc cells (J and M) rostral (H–J) and caudal (K–M) to the site of injury. Arrows indicate glycine-ir dorsal cells; arrowheads indicate glycine-ir lateral cells; barbed arrowheads indicate glycine-ir CSF-c cells; the star indicates the central canal. Dorsal is to the top and the midline to the right. In the graphs, each dot represents one animal and bars represent mean and 95% confidence interval. Abbreviations: R, rostral; C, caudal; D: dorsal interneurons; LAT: lateral interneurons. Scale bars: 40 μm. *0.01–0.05; **0.001–0.01; ***0.0001–0.001.

By means of glycine immunohistochemistry and confocal microscopy we quantified the long-term variations in the number of glycine-immunoreactive (-ir) neurons in the dorsal, lateral and cerebrospinal fluid-contacting (CSFc) cell populations of the spinal cord of larval sea lampreys, rostral and caudal to the level of a complete transection of the cord. Fig. 1 presents a scheme of a lateral view of a larva and its spinal cord (SC in the figure) indicating the site of injury and the regions of analysis, which were 150–300 μm rostral and caudal to the site of transection. In Fig. 2, we show representative photomicrographs of the spinal cord of larval lampreys revealing the glycine-ir neuronal populations at different time points after a complete injury of the cord, and graphs presenting the statistical analysis. Our data analyses show that glycine-ir cell numbers are recovered to control levels after the statistically significant acute loss of glycine immunoreactivity (Fig. 2). Table 1 shows the mean ± standard error of the mean (S.E.M.) number of glycine-ir neurons for each cell population and experimental group. The individual data from each animal is given in Supplementary File 1.

**Table 1:** Mean ± S.E.M. number of glycine-ir neurons for each cell population and experimental group.

		CSFc cell population	Dorsomedial population	Lateral population
Control		41.03 ± 5.78	56.31 ± 6.34	54.41 ± 8.32
1 HPL	Rostral	5.73 ± 3.41	7.74 ± 4.06	9.02 ± 5.3
	Caudal	0.74 ± 0.74	4.53 ± 3.01	5.13 ± 2.76
2 WPL	Rostral	55.5 ± 10.83	112 ± 13.47	77.05 ± 19.49
	Caudal	39.64 ± 8.36	74.64 ± 13.98	42.21 ± 6.84
4 WPL	Rostral	59.23 ± 10.09	98.25 ± 13.55	67.22 ± 7.48
	Caudal	52.31 ± 6.84	84.79 ± 12.26	65.98 ± 11.84
10 WPL	Rostral	37.36 ± 11.04	33.68 ± 11.03	48.18 ± 9.05
	Caudal	62.02 ± 9.63	75.75 ± 7.3	43.21 ± 6.47

## 2 Experimental design, materials, and methods

### 2.1 Animals

To obtain these data, we used larval sea lampreys (*Petromyzon marinus* L.; n = 33; body length: 100–140 mm; age: 5–7 years). Larvae of this age are developmentally stable and mature. We caught them in the river Ulla (Galicia, Northwest Spain) and kept them in aerated freshwater aquaria at 15 °C with a sand-bed river sediment until they were used for experimental procedures. Before the experiments, all animals were deeply anaesthetized with 0.1% tricaine methanesulfonate (MS-222; Sigma, St. Louis, MO) in lamprey Ringer solution (pH 7.4; NaCl 137 mM, KCl 2.9 mM, CaCl2 2.1 mM, HEPES 2 mM). The Bioethics Committee at the University of Santiago de Compostela and the *Consellería do Medio Rural e do Mar* of the *Xunta de Galicia* approved all the experiments leading to these data, which were performed in accordance to European Union and Spanish guidelines on animal care and experimentation.

### 2.2 Spinal cord injury surgical procedures

We randomly assigned the animals to any of these experimental groups: control un-lesioned animals (n = 9), and lesioned animals (with a complete spinal cord injury; SCI). We analysed the lesioned animals 1 hour post-lesion (hpl; n = 9), 2 weeks post lesion (wpl; n = 5), 4 wpl (n = 5) or 10 wpl (n = 5). We performed the complete SCI as previously described [2]. Briefly, starting from the dorsal midline, at the level of the 5th gill, we carefully cut the skin and muscle of the body wall until the SC was exposed. We held the body walls with home-made insect pin-hooks, and made a complete transection of the SC with Castroviejo scissors. We cut the SC in the transversal plane, visualizing the cut ends under the microscope to confirm the SC transection was complete. Immediately after the surgery, the animals were put on ice within two paper towels soaked in Ringer for 1 h. Then, we returned the animals to individual freshwater tanks and let them recover at 19.5 °C. Additionally, and once in the tanks, we checked that there was no movement below the site of injury.

### 2.3 Tissue processing

After the different recovery periods, we deeply anaesthetized the control and lesioned larvae and sacrificed them by decapitation. Then, we fixed the region of the body between the 4th and the 6th gills by immersion in 5% glutaraldehyde and 1% sodium metabisulfite (MB) in 0.05 M Tris-buffered saline (TBS; pH 7.4) for 20 h at 4 °C. Following fixation, we rinsed the tissue in 0.05 M TBS with 1% sodium metabisulfite (TBS-MB) several times during 6–8 h at 4 °C, and cryoprotected it in 30% sucrose in TBS overnight at 4 °C. Then, we embedded the tissue first in a 1:1 mix of 30% sucrose in TBS and Neg 50™ (Microm International GmbH, Walldorf, Germany) for 15 min, and then in Neg 50™. We froze the tissue in Neg 50™ using liquid nitrogen cooled isopentane, and finally, sectioned it on a cryostat (transverse plane; 14 μm thick).

### 2.4 Glycine immunofluorescence

First, we incubated the sections at 37 °C for 45 min to prevent the sections to wash off during the rinses. Then, we rinsed the sections in TBS-MB and subsequently pre-treated the sections with 0.2% NaBH4 in deionized water for 45 min to douse glutaraldehyde induced fluorescence, and we rinsed them again in TBS-MB. Following these steps, we incubated the sections with a rabbit polyclonal anti-glycine antibody (Immunosolution, Jesmond, Australia; 1: 3000; Cat# IG-1003; RRID: AB_10013221) in TBS-MB during 3 days at 4 °C. After rinsing in TBS, we incubated the sections for 1 h at room temperature with a Cy3-conjugated goat anti-rabbit immunoglobulin (Chemicon, Temecula, CA; 1:100; Cat# AP132C; RRID: AB_92489), rinsed them in TBS and mounted them with Mowiol. We used TBS (pH 7.4) containing 0.2% Triton X-100 and 15% normal goat serum to dilute the antibodies. We always performed the glycine immunofluorescence in parallel in sections of control un-lesioned and lesioned animals.

### 2.5 Anti-glycine antibody

Several assays have shown the specificity of the glycine antibody. The supplier raised the polyclonal anti-glycine antibody against a glycine-porcine thyroglobulin conjugate, and they tested it in sections of retina and cerebellum from various vertebrates. Furthermore, they performed dot blot immunoassays with a variety of amino acids found in proteins; the non-protein amino acids d-serine, d-alanine, and d-aspartate; GABA; and the glycine-containing tripeptide glutathione, which did not yield significant reactivity. Moreover, this antibody, developed by Dr. David V. Pow (University of Newcastle, Australia), has been used in several studies on glycinergic neurons of the central nervous system of the sea lamprey and other fishes [[3], [4], [5]].

### 2.6 Image acquisition

We photographed and analysed the sections with a spectral confocal microscope (model TCS-SP2; Leica, Wetzlar, Germany). We photographed 3 sections rostral and 3 sections caudal to the level of the 5th gill in control un-lesioned animals. In lesioned animals, we photographed 3 sections (1 of each 3 consecutive sections) within the 150–300 μm rostral and 3 sections (1 of each 3 consecutive sections) within the 150–300 μm caudal to the site of injury. We photographed only one random half of the SC for each of the sections from un-lesioned and lesioned animals. We took the microphotographs at 20× magnification (with a 1.5× digital zoom) without changing any of the microscope conditions to avoid the introduction of experimental variability. We employed the software LCS Lite (Leica) and Fiji (Image J, NIH, Bethesda, Maryland, USA, [6]) to process the stacks of confocal microphotographs, to generate a Z projection of the stack, and to compile a single tiff file of the microphotograph for the figures. Finally, we generated the figure plates and added the lettering with Adobe Photoshop CC 2018 (Adobe Systems, San José, CA, USA) and Corel Draw 2018 (Corel, Ottawa, Canada).

### 2.7 Quantification of glycine-ir cells

We quantified the three types of glycinergic cells found in the grey matter of the SC of lampreys: dorsal, lateral and cerebrospinal fluid-contacting (CSFc) glycine-ir cells. To obtain the raw data (Supplementary File 1), we quantified the cells manually by means of stereological counts of stacks of confocal microphotographs of the sections of the SC between 150 and 300 μm rostral and caudal to the site of injury (1 of each 3 consecutive sections). We counted the cells throughout the optical sections (one by one) using Fiji to avoid missing cells by possible cell overlapping. To keep track of the cells already counted in each optical section, we used the *Cell Counter* plug-in in Fiji (developed by Kurt de Vos, University of Sheffield). Then, we performed the stereological counting by disregarding the cells located in the first optical section of the stack of each section of the SC. We inferred the number of cells within the 150 μm comprised between 150 and 300 μm to the site of injury (rostral and caudal) from the number of cells counted in each spinal section. Finally, we calculated the mean number of cells for each cell population and animal and used these data for statistical analysis.

### 2.8 Statistical analysis

We used Prism 6 (GraphPad software, La Jolla, CA) to perform the statistical analysis. We present the data as mean ± S.E.M. in Table 1, and as mean and 95% confidence interval (CI) in Fig. 2. We analysed the data with a Kruskal-Wallis test and post-hoc Dunn's multiple comparisons test (significance level at 0.05). In Fig. 2, we present the significance values with a different number of asterisks: 1 asterisk (p value between 0.01 and 0.05), 2 asterisks (p value between 0.001 and 0.01) and 3 asterisks (p value between 0.0001 and 0.001).
